# A Novel Antimalarial Metabolite in Erythrocyte From the Hydroxylation of Dihydroartemisinin by *Cunninghamella elegans*

**DOI:** 10.3389/fchem.2022.850133

**Published:** 2022-04-26

**Authors:** Yue Bai, Yifan Zhao, Xinna Gao, Dong Zhang, Yue Ma, Lan Yang, Peng Sun

**Affiliations:** Institute of Chinese Meteria Medica, Artermisinin Research Center, Academy of Chinese Medical Sciences, Beijing, China

**Keywords:** dihydroartemisinin, microbial transformation, active metabolites in erythrocyte, hydroxylation, antimalarial activity

## Abstract

Dihydroartemisinin (DHA) is a sesquiterpene endoperoxide with prominent antimalarial efficacy, which was discovered by Professor Youyou Tu through the reduction of artemisinin in the 1970s. It is always a challenging work for scientists to investigate the metabolites of DHA in the red blood cells due to the complicated matrix background. As a bottleneck, the investigation of metabolites, especially exploring the pharmacodynamic material in the red blood cell, is necessary and significant for metabolism research of antimalarial agent. Recently, microbial transformation provides a green and economical means for mimicking mammal metabolism and synthesis active metabolites, based on which is one efficient route for drug discovery. In this study, a strain from *Cunninghamella* was employed as an efficient tool to explore active metabolites of DHA in erythrocyte. Microbial transformation products of DHA by *Cunninghamella elegans* CICC 40250 were detected and analyzed by ultra-performance liquid chromatography (UPLC)-electrospray ionization (ESI)-quadrupole time-of-flight (Q-TOF)-mass spectrometry (MS^E^), and the main products were isolated and identified. The antimalarial activity of the isolated products was also screened *in vitro*. Totally, nine products were discovered through UPLC-ESI-QTOF-MS^E^, and three main products with novel chemical structures were isolated for the first time, which were also detected in red blood cells as the metabolites of DHA. After evaluation, 7*β*-hydroxydihydroartemisinin (**M1**) exhibited a good antimalarial activity with an IC_50_ value of 133 nM against *Plasmodium falciparum* (Pf.) 3D7. The structure and stereo-configuration of novel compound **M1** were validated *via* X-ray single crystal diffraction. Microbial transformation was firstly employed as the appropriate model for metabolic simulation in erythrocyte of DHA. Three novel metabolites in erythrocyte were obtained for the first time through our microbial model, and one of which was found to show moderate antimalarial activity. This work provided a new research foundation for antimalarial drug discovery.

## Introduction

Dihydroartemisinin (DHA), a famous derivative of artemisinin (Qinghaosu, ART) through C-10 reduction of lactone carbonyl by sodium borohydride, was discovered by Youyou Tu in 1973 (Tu, 2009; Tu, 2016). Owing to their excellent antimalarial activity compared to artemisinin, DHA and its derivatives have been employed as the first-line drugs to treat malarial for several decades (World Health Organization, 2020). Apart from the application in malarial treatment, DHA was also found to exhibit a broad scope of biological activities, including anti-inflammatory, antitumor, antiviral, and immunoregulatory activities (Wang et al., 2018; Li et al., 2019; Zhang et al., 2020). Given the promising therapeutic potential in clinical, the metabolic study of DHA in the body is of great interest to researchers. Although considerable efforts have been devoted to the research on the disposal process of DHA, and a series of metabolites in body have been identified, the study on the metabolites of DHA in erythrocytes remains rarely involved but challenging. Indeed, the exploration of active metabolites in erythrocyte is of considerable important for antimalarial drug because red blood cells are the actual sites where the plasmodium lives and the drug works. Followed by Prof. Tu, our team has been engaged in the metabolism study of ART, DHA, and its derivatives in the last few years (Tu, 2011; Yang and Zhang, 2017; Ma et al., 2019a; Ma et al., 2019b; Bai et al., 2019; Zhao et al., 2021).

Recently, the microbial transformation has become an efficient tool for metabolic simulations of drugs (Parshikov et al., 2004). It exhibited good function for molecular modification and synthesis active metabolites environmental friendly and cheaper cost. Filamentous fungi, which share the similar cytochrome P450 enzymes with human, have been proved to possess the ability to mimic the metabolic process in body (Asha and Vidyavathi, 2009). To get some insight of the *in vivo* process of DHA, some projects have been conducted through filamentous fungi-mediated strategy, and six derivatives of DHA including 14-hydroxymethyl dihydroartemisinin (Hu et al., 1991), 9*α*-hydroxyartemethin-I (Han and Lee, 2016), 3*α*-hydroxydeoxydihydroartemisinin, 8*α*-hydroxydeoxyartemisinin, deoxyarte-misinin, and 9*α*-hydroxydihyartemisinin (Wang et al., 2013) have been obtained. Unfortunately, there are no scientific results to validate whether these compounds are consistent with mammalian metabolites. The inhibitory efficiency on plasmodium of these compounds has never been studied.

**SCHEME 1:**
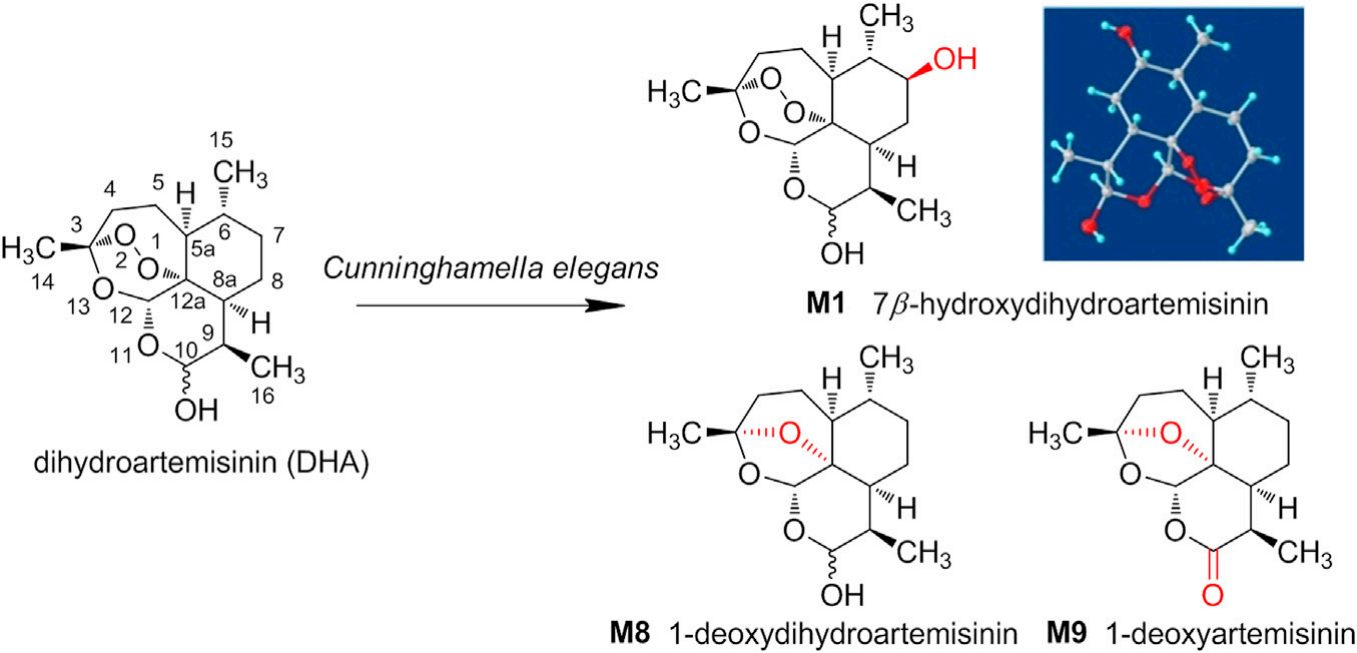
The structures of dihydroartemisinin (DHA) and its derivates from *Cunninghamella elegans*.

Herein, the metabolic study of DHA was performed employing *Cunninghamella elegans* (*C. elegans*) CICC 40250, and the products were analyzed and compared with the metabolites of DHA in erythrocytes. As a result, from microbial transformation, a total of nine DHA derivatives (**M1**–**M9**) were detected by UPLC-MS and three were isolated, including 7*β*-hydroxydihydroartemisinin (**M1**), 1-deoxydihydroartemisinin (**M8**), and 1-deoxyartemisinin (**M9**). The three gained products were also identified in erythrocytes. The structure and stereo-configuration of the novel product (**M1**) were validated via X-ray single crystal diffraction, and reported here for the first time. Moreover, **M1** was also proven to exhibit *in vitro* antimalarial activity against Pf 3D7, with an IC_50_ value of 133 nM. As well as we know, this is the first attempt to isolate and identify the metabolites of DHA in red blood cells via microbial transformation. The discovery and synthesis of antimalarial products provide the possibility for further drug development by eco-friendly microbial transformation. The structures of DHA and its derivates from *C. elegans* were shown in Scheme 1.

## Materials and Methods

### Materials and Reagents

DHA was obtained from the Kunming Pharmaceutical Group (Chongqing, China, batch number C00220160402). The compound had purity ≥ 99%. Methanol, acetonitrile, and formic acid were HPLC grade and purchased from Fisher (Geel, Belgium). Other chemicals in this work were of analytical grade and purchased from Beijing Chemical Works (Beijing, China). Silicone filler, silica gel GF_254_ thin layer plates, and silica gel GF_254_ thin layer preparation plates were purchased from Qingdao Marine Chemical Group Corporation (Qingdao, China). Silica gel (200–300 mesh, Qingdao Marine Chemical Group Corporation, Qingdao, China), Silica Flash Column 330 g, and Chormatorex (FujiSilysia Chemical, Japan) were used for column chromatography. Water was prepared using a Milli-Q system operating at 18.2 MΩ (Millipore, Bedford, MA, United States). All other chemicals used were purchased from Fisher Scientific or Beijing Chemical Works, and were of the highest purity available.

The UPLC-ESI-Q-TOF-MS^E^ system consisted of a Waters ACQUITY I-class UPLC and Xevo G2-XS Q-TOF Mass Spectrometer (Waters, Manchester, United Kingdom). ^1^H (600 MHz), ^13^C (150 MHz), and NMR spectra were recorded on a Bruker AV 600 spectrometer (Bruker Corporation, Fallanden, Switzerland) with TMS as an internal reference. X-ray diffraction experiments were performed using a Bruker D8 venture diffractometer with Cu Kα radiation. (Crystals of metabolites were obtained from EtOAc and acetone by slow evaporation of solvent at room temperature.)

### Microorganism and Biotransformation Procedure

*C. elegans* CICC 40250 (**MT1**) was obtained from the China Center of Industrial Culture Collection (Beijing, China). Culture and biotransformation experiments were conducted in a medium composed of 20 g Sabouraud dextrose broth (Oxoid, Basingstoke, United Kingdom), 10 g peptone (Solarbio, Beijing, China), 15 g sucrose (Solarbio, Beijing, China), and 1,000 ml deionized water.

Two-stage fermentation was employed in this study (Zhan et al., 2017). The substrate was dissolved in MeOH at a concentration of 25 mg/ml, and 2 ml of the solution was added into each flask after the second fermentation stage, producing a DHA concentration of 0.5 mg/ml. Cultures were incubated at 28°C and shaken at 180 rpm for 14 days. Mycelia and broth were then separated by filtration. The filtrate was extracted with EtOAc (1:1, v/v) three times, and the extract was evaporated under vacuum to produce a brown residue.

### UPLC–MS^E^ Conditions and Data Processing

The UPLC-ESI-QTOF-MS^E^ system consisted of a Waters ACQUITY I-class UPLC and Xevo G2-XS Q-TOF mass spectrometer equipped with electrospray ionization (ESI) source. Chromatographic separation was achieved using an ACQUITY UPLC BEH C18 (2.1 mm × 100 mm, 1.7 µm, Waters). The mobile phase consisted of solvent A (H_2_O containing 0.1% formic acid, v/v) and solvent B (acetonitrile containing 0.1% formic acid, v/v). The gradient program for biosamples included three segments: 5–100% B from 0 to 15 min; followed by 100–5% B from 15 to 17 min; and a postrun of 3 min for column equilibration. The flow rate was 0.4 ml/min, and the temperature was 30°C throughout the analysis.

The MS was operated in positive ionization mode across a scan range of m/z 50 to 1,000, with a scan time of 0.2 s. Source parameters: source temperature 150°C, cone gas 50 L/h, desolations temperature 450°C, and desolation gas flow 800 L/h. Argon (99.95%) was used for collision-induced dissociation, and N_2_ was used as the drift gas. The low collision energy was set to 6 eV, and the high collision energy was ramped from 12 to 25 eV. MSE analysis was employed for simultaneous acquisition of the exact mass of small molecules at high and low collision energies.

All data processing was performed using UNIFI 1.9 (Waters, Manchester, United Kingdom). Components were identified using the following 3D peak detection features: low-energy limits of 150 and high-energy limits of 20, isotope clustering, and high-to-low energy association within a 0.5 fraction of the chromatographic and drift peak width, with a mass accuracy of ±2 mDa. The maximum number of allowed fragment ions per match was set to 10.

### Extraction and Isolation of Transformed Products

Scale-up fermentations were also carried out according to the standard two-stage procedure. Under the same temperature-controlled shaking conditions, each 2000 ml flask contained 500 ml Sabouraud glucose liquid medium, and DHA (6 g) was dissolved in MeOH at a concentration of 25 mg/ml and evenly distributed in all flasks. After incubation for 14 days, mycelia and broth were separated by filtration. The filtrate was extracted with EtOAc (1:1, v/v) three times, and the extract was evaporated under vacuum to produce a brown residue. The EtOAc extract was fractionated by a Silica Flash Column using a gradient solvent system of petroleum ether-EtOAc to afford 43 fractions (Fr. 1-Fr. 43) according to TLC analysis.

Fr. 15 (0.5 g) was subjected to a Chromatorex column and eluted with a gradient solvent system of petroleum ether-EtOAc to afford compound **M1** (100 mg). Fr. 30 was separated by recrystallization to obtain compound **M9** (20 mg). Fr. 40 (1 g) was separated over a CHORMATOREX column and eluted with a gradient solvent system of petroleum ether-acetone to give 14 subfractions. Fr. 40-2 was subjected to thin layer preparation plates to obtain compound **M8** (80 mg).

### Evaluation of Antimalarial Activity

Compounds **M1**, **M8**, and **M9** were diluted in DMSO and examined for antimalarial activity *in vitro* against the *Pf*. 3D7 clone. Artemisinin was used as a positive control drug. Inhibition of plasmodium proliferation by different concentrations of transformed products was determined according to a certain formula, and the *in vitro* antimalarial activity of the drug was evaluated with the IC_50_.

## Results

### Analysis of the Microbial Transformation DHA Products From *C. elegans* 40250

**FIGURE 1:**
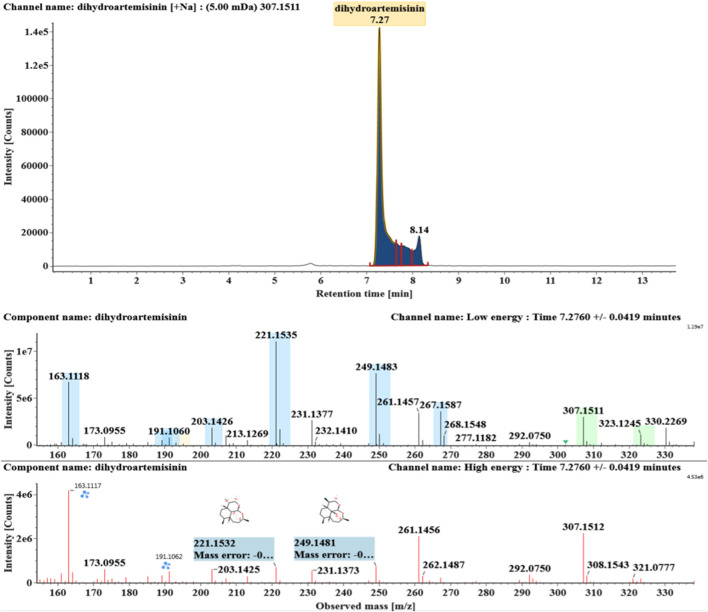
Extracted ion chromatograms and mass spectrometry (MS^E^) spectra of DHA.

**FIGURE 2:**
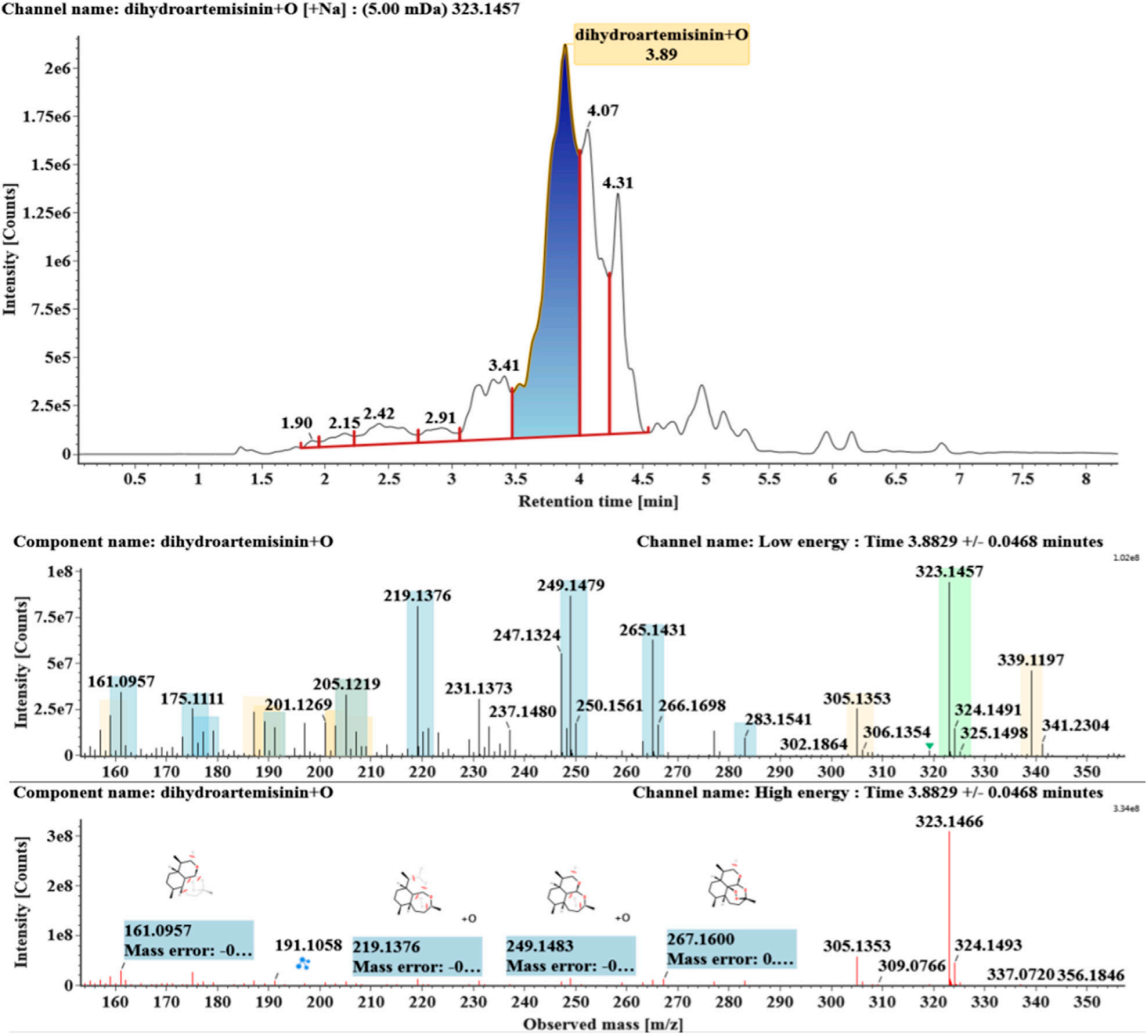
Extracted ion chromatograms and mass spectrometry (MS^E^) spectra of **M1**.

**FIGURE 3:**
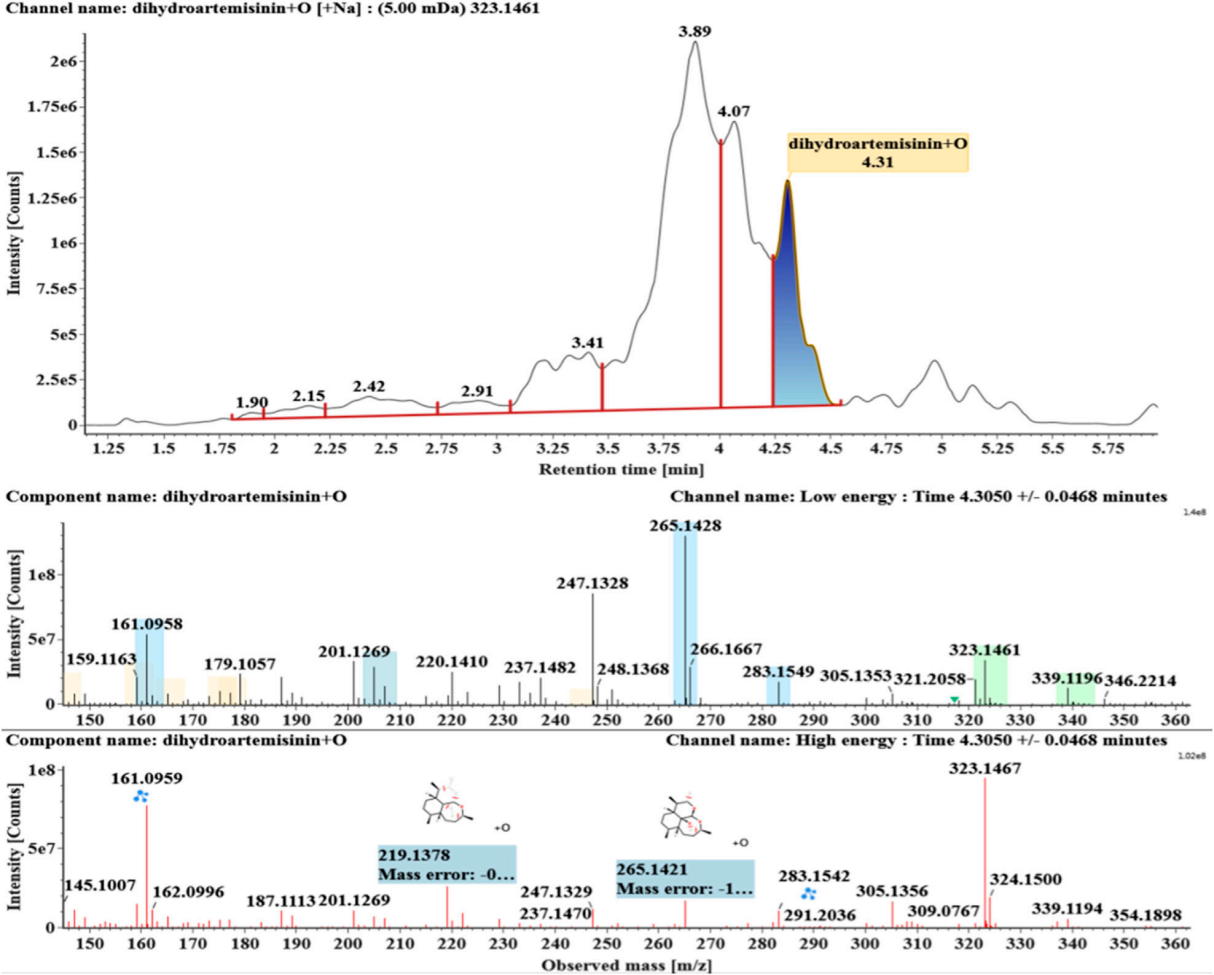
Extracted ion chromatograms and mass spectrometry (MS^E^) spectra of **M2**.

**FIGURE 4:**
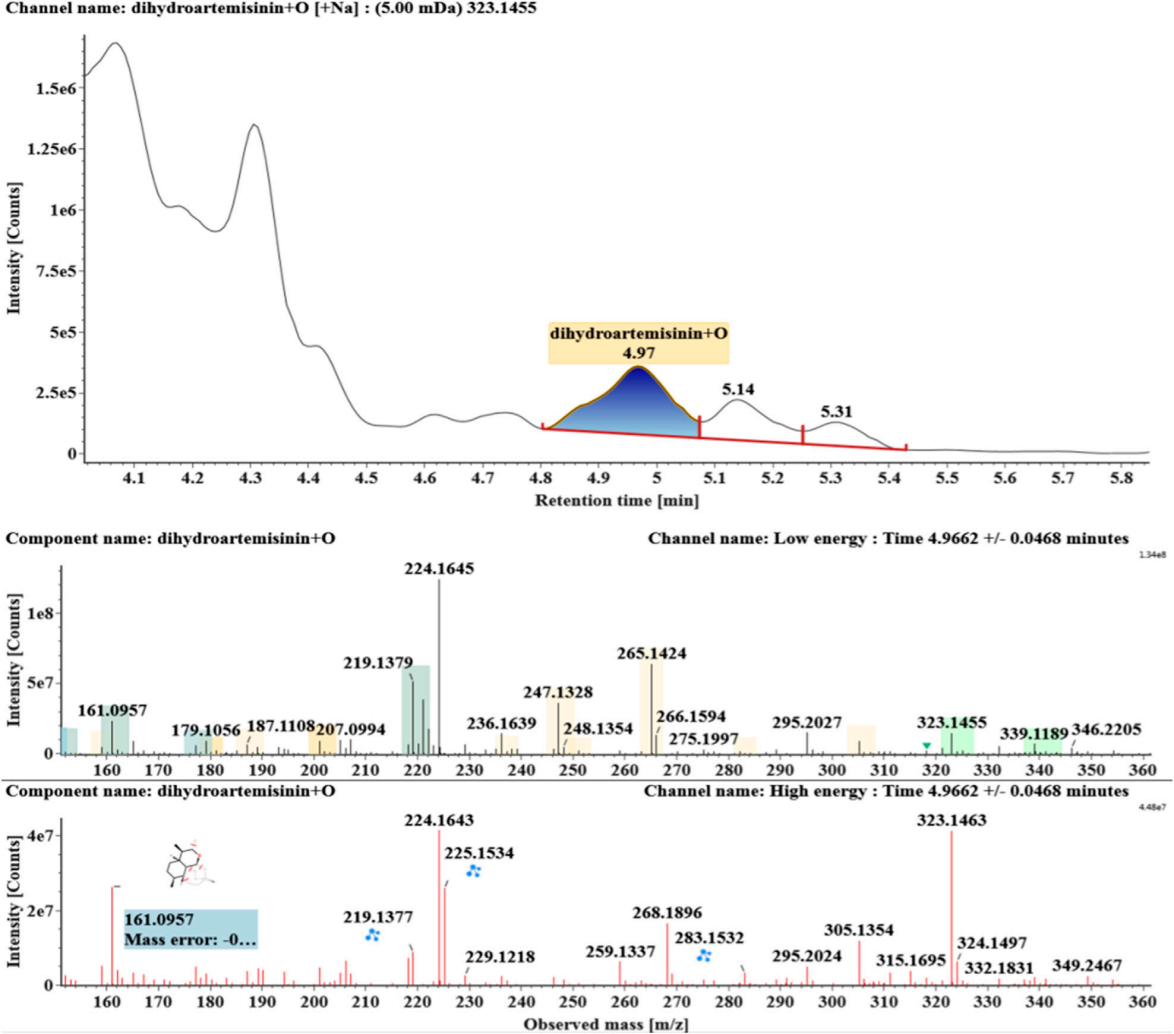
Extracted ion chromatograms and mass spectrometry (MS^E^) spectra of **M3**.

**FIGURE 5:**
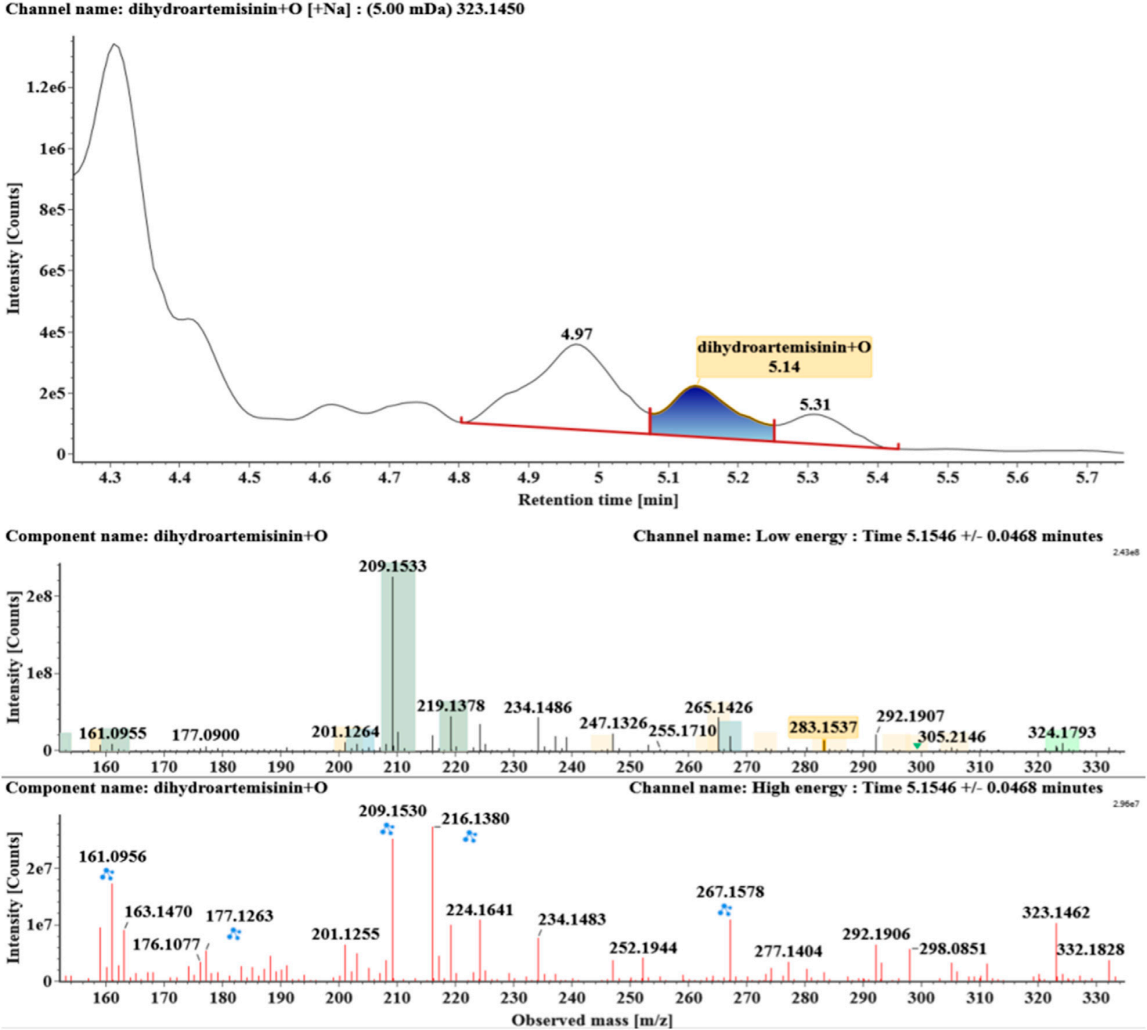
Extracted ion chromatograms and mass spectrometry (MS^E^) spectra of **M4**.

**FIGURE 6:**
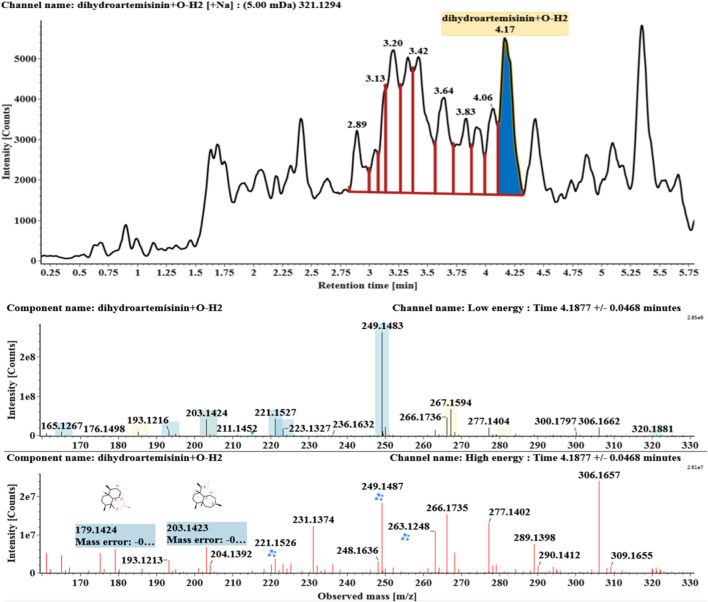
Extracted ion chromatograms and mass spectrometry (MS^E^) spectra of **M5**.

**FIGURE 7:**
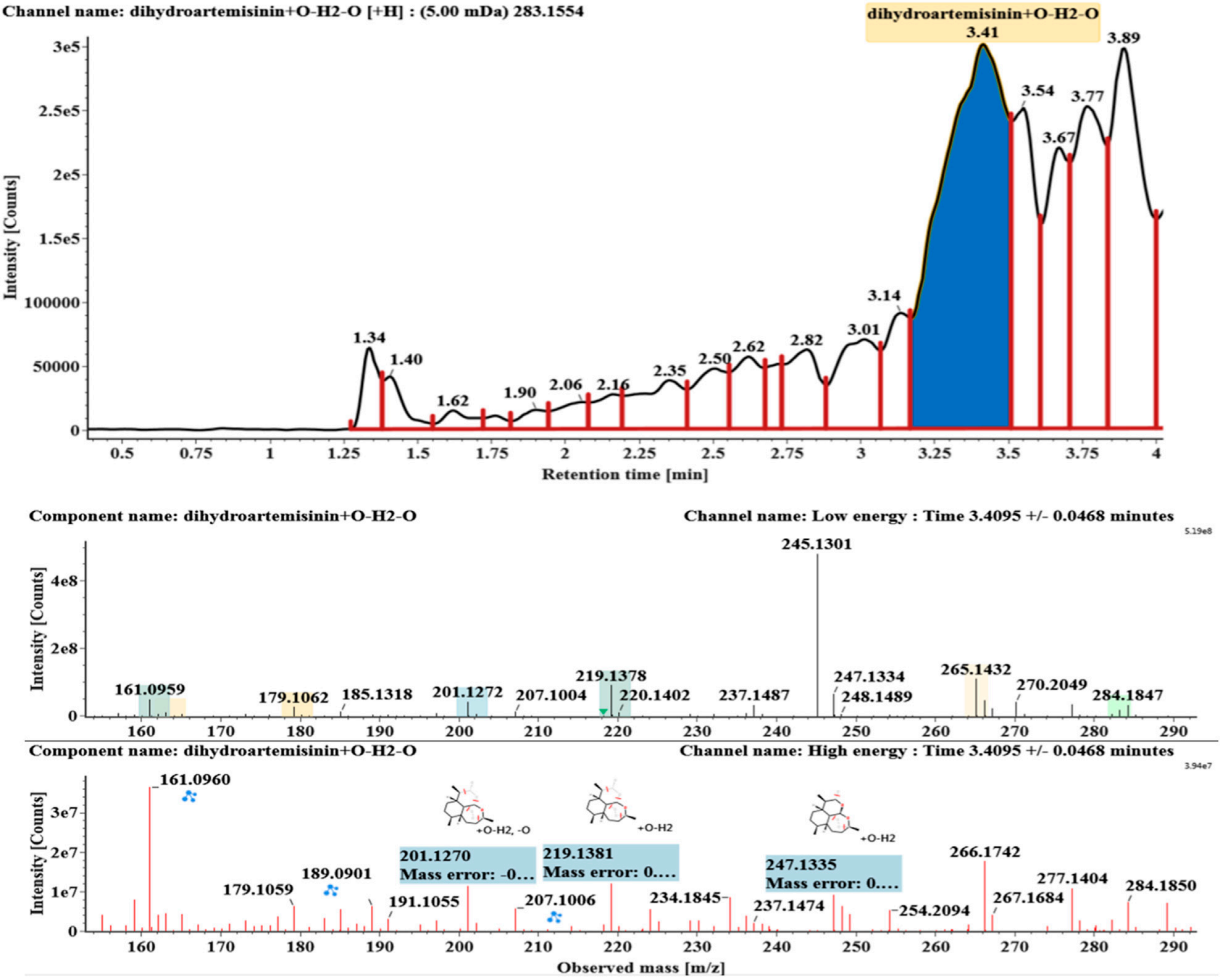
Extracted ion chromatograms and mass spectrometry (MS^E^) spectra of **M6**.

**FIGURE 8:**
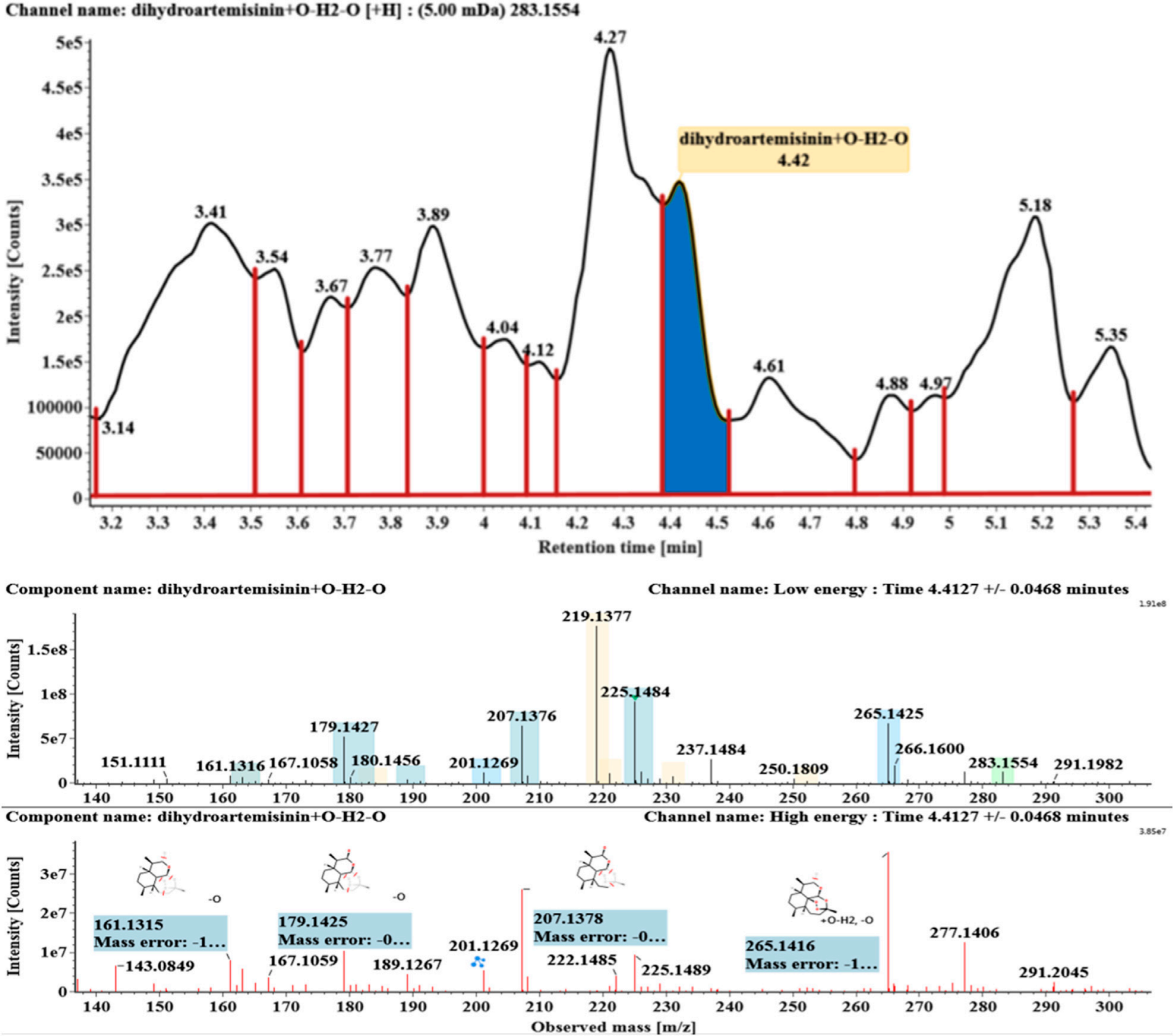
Extracted ion chromatograms and mass spectrometry (MS^E^) spectra of **M7**.

**FIGURE 9:**
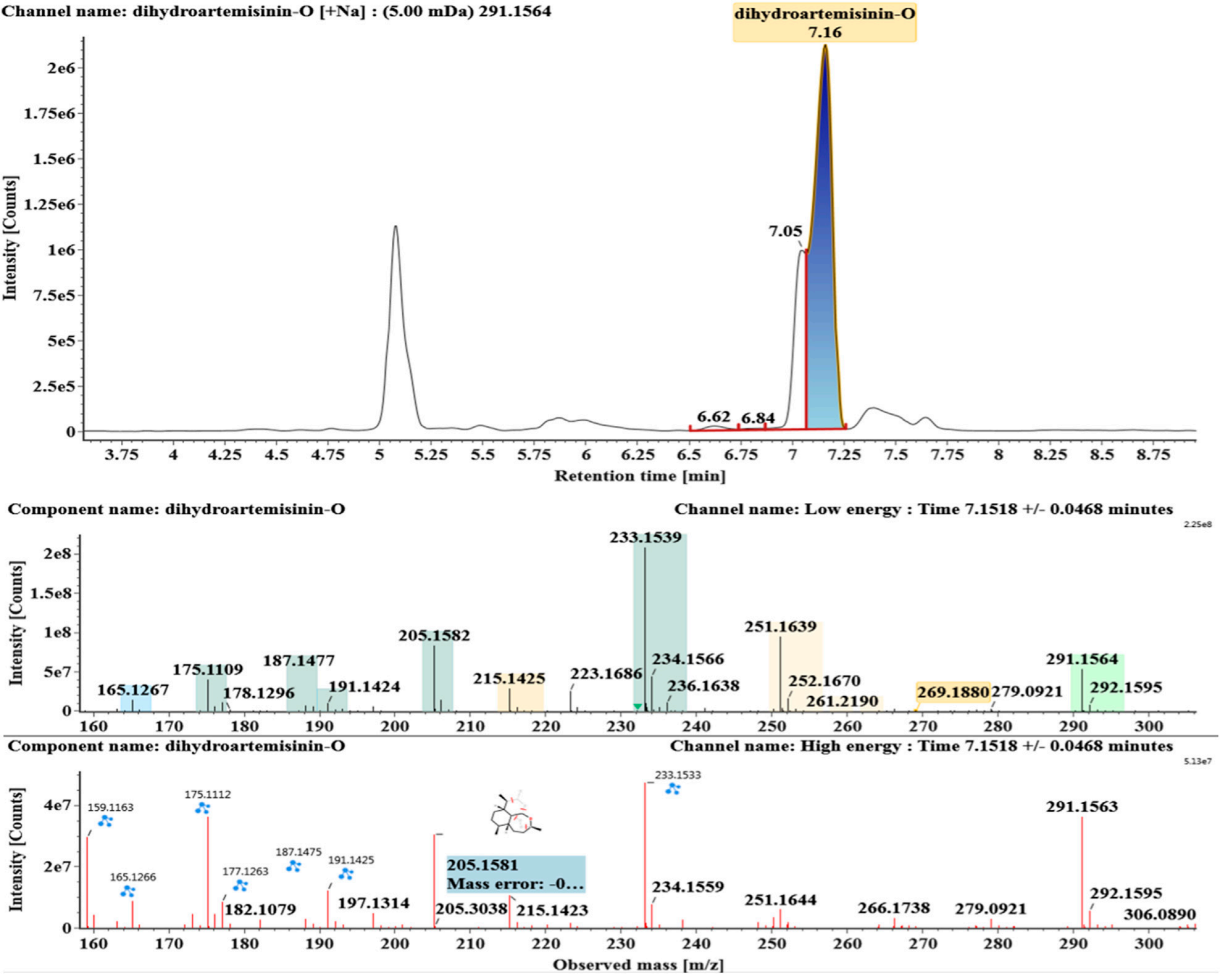
Extracted ion chromatograms and mass spectrometry (MS^E^) spectra of **M8**.

**FIGURE 10:**
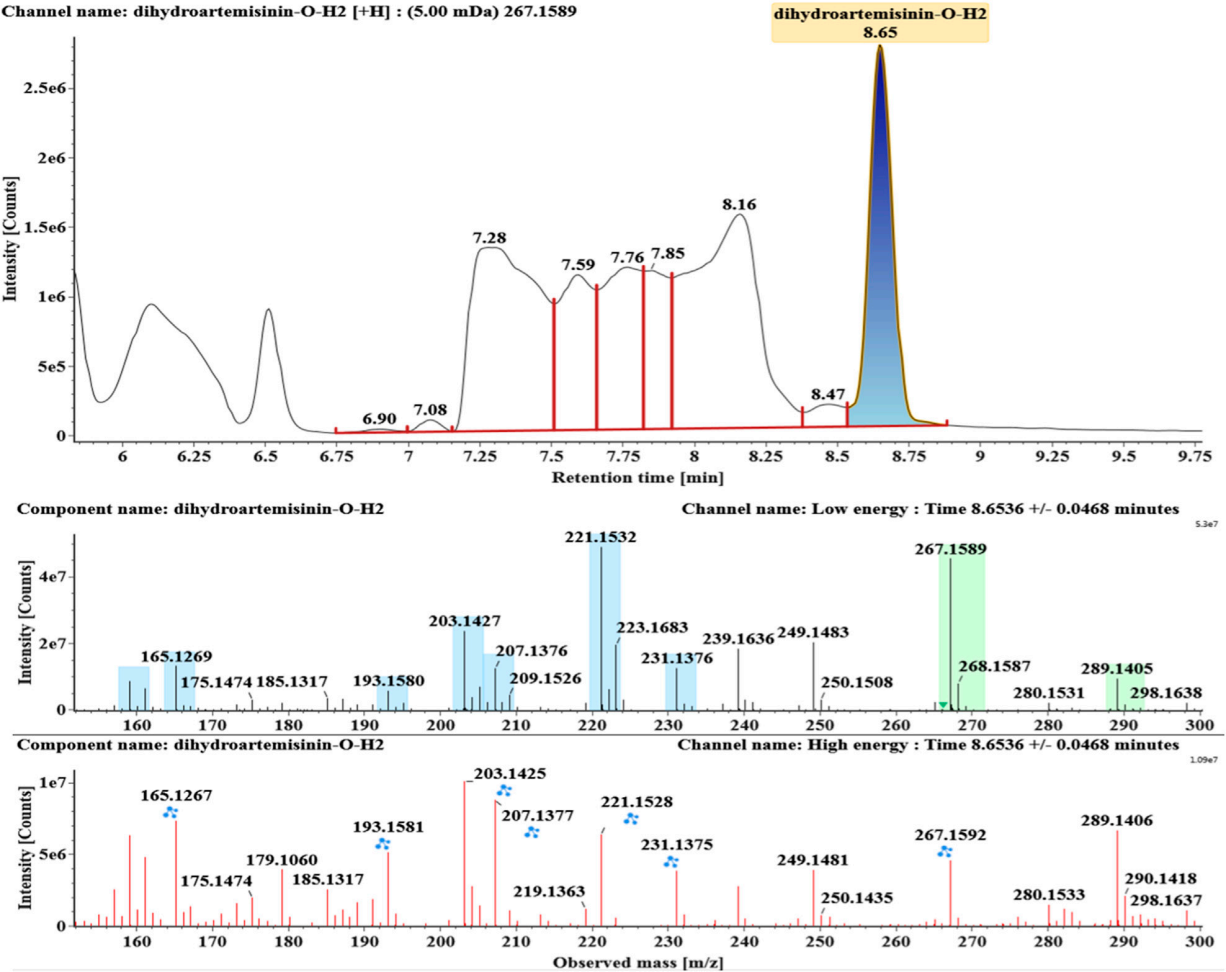
Extracted ion chromatograms and mass spectrometry (MS^E^) spectra of **M9**.

The microbial transformation products were identified with UNIFI platform based on chromatographic and mass spectral characteristics provided by reference substances DHA, such as retention time, exact mass, quasi-molecular ions, in-source fragments, characteristic fragments. A total of nine products were found from *C. elegans* 40250, classified into hydroxylated DHAs (DHA + O), hydroxylated and dehydrogenated DHAs (DHA + O-H2), hydroxylated and dehydrated DHAs (DHA + O-H2-O), deoxygenated DHA (DHA-O), and dehydrated DHA (DHA-H2-O). All identified products were listed in Table 1. Extracted ion chromatograms and MS^E^ spectra of DHA biotransformation products were shown in Figure 1. Also extracted ion chromatograms and MS^E^ spectra of its products were shown in Figures 2–10, respectively. The response strength of nine products, which could partly estimate the transform yield of compounds, is shown in Supplementary Figure S1.

**TABLE 1:** Summary of microbial transformation products of dihydroartemisinin.

No	Component	Formula	Observed *m/z*	RT (min)	Major fragments	Response
M1	DHA + O	C_15_H_24_O_6_+Na^+^	323.1457	3.89	323, 283, 265, 249, 247, 231, 219	1709644
M2	DHA + O	C_15_H_24_O_6_+Na^+^	323.1461	4.31	339, 323, 283, 265, 247, 237, 219	613851
M3	DHA + O	C_15_H_24_O_6_+Na^+^	323.1455	4.97	339, 323, 283, 265, 247, 219	267107
M4	DHA + O	C_15_H_24_O_6_+Na^+^	323.1450	5.14	323, 283, 265, 247, 219	121182
M5	DHA + O-H2	C_15_H_22_O_6_+Na^+^	321.1294	4.17	321, 267, 249, 231, 221, 203	3365
M6	DHA + O-H2O	C_15_H_22_O_5_+H^+^	283.1554	3.41	283, 265, 247, 237, 219, 201	333881
M7	DHA + O-H2O	C_15_H_22_O_5_+H^+^	283.1554	4.42	283, 265, 237, 225, 219, 201	220113
M8	DHA-O	C_15_H_24_O_4_+Na^+^	291.1564	7.16	291, 269, 251, 233, 223, 205, 187	926704
M9	DHA-H2O	C_15_H_22_O_4_+H^+^	267.1589	8.65	289, 267, 249, 231, 221, 203, 185	751976

DHA, the reference substance, was eluted at 7.27 min. Its molecular ion [M + Na]^+^ was observed at *m/z* 307.1509. The fragment ions at *m/z* 267.1590 and 249.1485 were generated by the successive loss of water from *m/z* 307.1509. The fragment ions at *m/z* 221.1538 represented the loss of HCOOH from *m/z* 267.1590. The fragment ions at *m/z* 203.1432 resulted from the loss of water from *m/z* 221.1538. Extracted ion chromatograms and mass spectrometry (MS^E^) spectra of DHA were shown in Figure 1.

Hydroxylated DHAs (DHA + O, **M1-M4**) were detected at 3.89, 4.31, 4.97, and 5.14 min. Their molecular ions [M + Na]^+^ were observed at *m/z* 323.1457, 323.1461, 323.1455, and 323.1450, respectively. They exhibited the same fragment ions. The fragment ions at *m/z* 283, 265, and 247 were generated by the successive loss of water from *m/z* 323. The fragment ions at *m/z* 219 represented the loss of HCOOH from *m/z* 265. The fragment ions at *m/z* 201 resulted from the loss of water from *m/z* 219. Extracted ion chromatograms and mass spectrometry (MS^E^) spectra of **M1**-**M4** were shown in Figures 2–5, respectively.

Hydroxylated and dehydrogenated DHA (DHA + O-H2, **M5**) was detected at 4.17 min. Its molecular ion [M + Na]^+^ was observed at *m/z* 321.1294. The fragment ions at *m/z* 249 and 231 were generated by the successive loss of water from *m/z* 267. The fragment ions at *m/z* 221 represented the loss of HCOOH from *m/z* 267 or the loss of CO from *m/z* 249. The fragment ions at *m/z* 203 resulted from the loss of water from *m/z* 221. Extracted ion chromatograms and mass spectrometry (MS^E^) spectra of **M5** were shown in Figure 6.

Hydroxylated and dehydrated DHAs (DHA + O-H2O, **M6-M7**) were detected at 3.41 and 4.42 min, respectively. The molecular ions [M + H]^+^ were observed at *m/z* 283.1554. Consistent with DHA, these products showed a series of fragment ions resulting from the loss of H2O, CO, or HCOOH, such as *m/z* 265, 247, 237, 219, and 201. The fragment ions at *m/z* 265 and 247 were generated by the successive loss of water from *m/z* 283. The fragment ions at *m/z* 237 represented the loss of CO from *m/z* 265. Extracted ion chromatograms and mass spectrometry (MS^E^) spectra of **M6** and **M7** were shown in Figures 6–8, respectively.

Deoxygenated DHA (DHA-O, **M8**) and dehydrated DHA (DHA-H2O, **M9**) were detected at 7.16 and 8.65 min, respectively. Their molecular ions [M + Na]^+^ were observed at *m/z* 291.1564 and *m/z* 289.1405, respectively. They showed similar fragment ions except with a 2 Da (a hydrogen atom) mass shift, such as *m/z* 251, *m/z* 249, *m/z* 233, *m/z* 231, *m/z* 205, *m/z* 203, *m/z* 187, and *m/z* 185. The fragment ions at *m/z* 251 and *m/z* 249 were generated by the successive loss of water from *m/z* 291 and *m/z* 289, respectively. The fragment ions at *m/z* 205 and *m/z* 203 represented the loss of CO from *m/z* 233 and *m/z* 231, respectively. Their ions exhibited the same mechanism resulting from the loss of CO or H2O. Extracted ion chromatograms and mass spectrometry (MS^E^) spectra of **M8** and **M9** were shown in Figures 9 and 10, respectively.

Furthermore, as a semiquantitative comparative chromatography analysis, the relative contents of modified dihydroartemisinins (**M1–M9**) were evaluated by mass spectral response abundance data. As a result, **M1** had the highest content of all compounds, followed by **M8** and **M9**. And the numerical results of response provided the guidance for further isolation. The histogram of the response of all DHA products is shown in Supplementary Figure S1.

### Structural Elucidation of the Compounds Isolated From MT1

**SCHEME 2:**
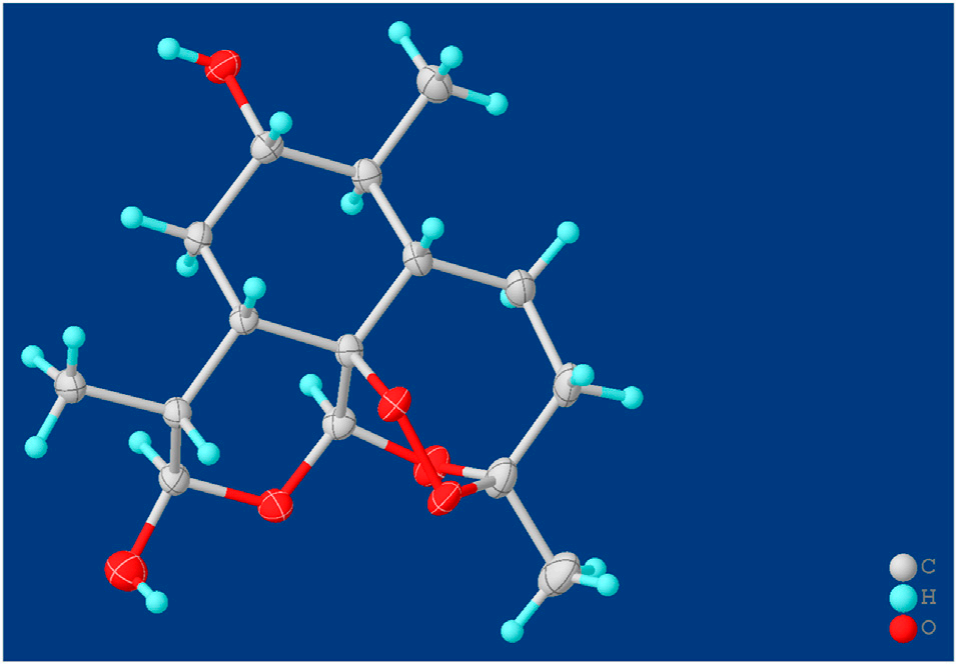
X-ray crystal structures of compound **M1**.

Compound **M1** colorless needles (from EtOAc and acetone). ESI-HRMS calcd. for [M + Na]^+^, C_15_H_24_O_6_Na, 323.1471; found *m/z* 323.1457. ^1^H and ^13^C NMR data are shown in Table 2. The HSQC spectrum showed correlations of H-12 (*δ* 5.39) with C-12 (*δ* 90.4), C10-OH (*δ* 4.64) with C-10 (*δ* 93.7), and H-7 (*δ* 2.96) with C-7 (*δ* 72.6), which supported compound **M1** as a hydroxylation product. The HMBC spectrum showed correlations of C10-OH (*δ* 4.64) with C-8a (*δ* 43.9), C-7 (*δ* 72.6) and C-8 (*δ* 31.2), along with the correlations of C-7 (*δ* 72.6) with H-14 (*δ* 0.94) and H-15 (*δ* 1.28). These spectral dates suggested that the hydroxylation occurred at C-7. Moreover, the absolute configuration of the hydroxylated group in the stereogenic carbon center was determined to be *β*-OH by single-crystal X-ray diffraction analysis (Scheme 2). A single crystal was obtained from EtOAc and acetone by slow evaporation of the solvent at room temperature. A colorless block-shaped crystal with dimensions of 0.20 × 0.15 × 0.10 mm^3^ was mounted. Data were collected using a Bruker APEX-II CCD diffractometer operating at T = 150.0 K and measured and scans using CuKa radiation. The maximum resolution achieved was = 72.120° (0.81 Å). The final completeness is 98.90% out to 72.120° in. Crystal data: C_15_H_24_O_6,_ Mr = 300.34, triclinic, P1, a = 9.6563 (6) Å, b = 9.9540 (6) Å, c = 15.6044 (9) Å, *α* = 93.832 (3)°, *β* = 90.860 (3)°, *γ* = 102.948 (3)°, V = 1,457.78 (15) Å3, Z = 4, Z' = 4, (CuKα) = 0.875, 28521 reflections measured, 10327 unique (Rint = 0.0363), which were used in all calculations. The final wR2 was 0.1013 (all data), and R1 was 0.0383 (I ≥ 2 (I)). Crystallographic data of compound **M1** have been deposited to CCDC (http://www.ccdc.cam.ac.uk, No. CCDC 2039245). Thus, the structure of compound **M1** was elucidated as 7*β*-hydroxydihydroartemisinin.

**TABLE 2:** ^1^H NMR (600 MHz) and^13^C NMR (150 MHz) data of three compounds (*δ*: ppm).

	Compound **M1** (DMSO-*d*_*6*_)		Compound **M8** (CDCl_3_)		Compound **M9** (CDCl_3_)	
Position	*δ*_*H*_ (*J* in Hz)	*δ*_*C*_	*δ*_*H*_ (*J* in Hz)	*δ*_*C*_	*δ*_*H*_ (*J* in Hz)	*δ*_*C*_
3		103.67		107.08		108.20
4		36.48	1.56 (m)	33.27	1.72 (t),1.56 (m)	32.96
5		24.78	1.65 (m)	21.05	1.84 (d)	21.01
5a	1.31-1.35 (dd, 23,13)	49.46		44.65		43.61
6	1.55-1.58 (m)	43.17		34.34		34.34
7	2.96 (s)	72.60	1.14 (m)	33.57	1.72 (t), 1.04 (m)	32.44
8		31.22		21.78	1.84 (d), 0.94 (t)	22.51
8a		43.87		40.34	1.94 (d)	41.41
9		33.97	2.26 (m)	33.00	3.12 (m)	31.75
10	4.64 (s)	93.74		95.99		170.82
12	5.39 (s)	90.41	5.28 (d)	95.13	5.63 (s)	98.62
12a		80.18		81.44		81.38
13	1.28 (s)	26.11	0.94 (d)	23.37	1.46 (s)	22.96
14	0.94 (d)	15.95	0.83 (d)	17.77	0.87 (d)	17.57
15	0.78 (d, 6.3)	13.30	1.47 (s)	13.93	1.13 (d)	11.61
10-OH	6.41 (d, 6.8)		4.71 (t, 6.4)			
7-OH	4.64 (d, J = 4. 9 Hz)					

Compound **M8** white powder (EtOAc). ESI-HRMS calcd. for [M + Na]^+^, C_15_H_24_O_4_Na, 291.1572; found *m/z* 291.1564. ^1^H and ^13^C NMR data are shown in Table 2. The data supported the compound **M8** was 1-deoxydihydroartemisinin (Han and Lee, 2017).

Compound **M9** white powder (EtOAc). ESI-HRMS calcd. for [M + H]^+^, C_15_H_22_O_4_H, 267.1596; found *m/z* 267.1589. HR-ESI-MS: [M + H]^+^ at *m/z* at 267.1589 (calcd. for C_15_H_22_O_4_). ^1^H and ^13^C NMR data are shown in Table 2. The data supported the compound **M9** was 1-deoxyartemisinin (Lee et al., 1989).

### Compared the Products M1, M8, and M9 With Metabolites in Erythrocytes

**FIGURE 11:**
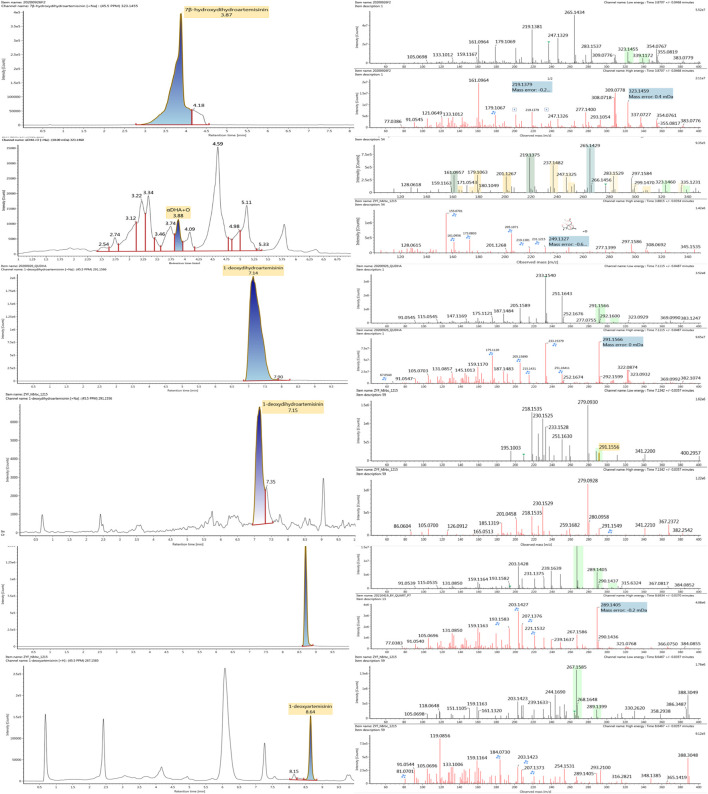
Extracted ion chromatograms and mass spectrometry (MS^E^) spectra of products **M1**, **M8**, and **M9** and the metabolites of DHA in red blood cells.

Based on the analysis results of DHA metabolites *in vivo*, the three products from *C. elegans* 40250 were compared with *in vivo* metabolites by UPLC-ESI-Q-TOF-MS^E^. The retention time and the mass behavior, such as exact mass, quasi-molecular ions, in-source fragments, and characteristic fragments, are compared to confirm the consistency. The results revealed that **M1**, **M8,** and **M9** were the common metabolites of DHA in erythrocyte. The extracted ion chromatograms and mass spectrometry (MS^E^) spectra of products **M1**, **M8,** and **M9** and the metabolites of DHA in red blood cells were shown in Figure 11.

### Antimalarial Activity *in vitro*

The positive control drug artemisinin exhibited *in vitro* antimalarial activity against *Pf.* 3D7 with an IC_50_ (50% inhibition concentration) value of 11 nM. Compound **M1** was indicated to exhibit *in vitro* antimalarial activity against *Pf*. 3D7 with an IC_50_ value of 133 nM. Compound **M8** and **M9** showed no activity against *Pf*. 3D7.

## Discussion

As known, considering the low concentrations and complex detection background, it is a bottleneck for exploring the drug metabolism in erythrocytes. Drugs accumulated in cells are difficult to meet the demand for analysis and isolation. Compared with the traditional metabolism research model *in vitro*, microbial transformation can mimic mammal metabolic action according to the similar CYP 450s in microorganisms with the advantage of metabolites accumulation eco-friendly and affordably. Herein, the *C. elegans* was chosen as an applicable model *in vitro* for studying DHA metabolism in red blood cells based on the previous research foundation (the experimental data were unpublished). Artemisinin and its derivatives are the first-line antimalarial agents for saving millions of people’s life. There are four drugs derived from artemisinin, including DHA, artemether, arteether, and artesunate. Given the IC_50_ value of antimalarial activity against *Pf.* 3D7 *in vitro*, the derivatives showed better antimalarial activity than artemisinin, at 4–6 nM approximately, while the value of artemisinin is at 12 nM (Kreidenweiss et al., 2008; Reiter et al., 2015). In this work, the antimalarial metabolite **M1** exhibited less active against *Pf.* 3D7 *in vitro*, for about a 10th of the activity of artemisinin and a 20th of DHA. However, the antimalarial mechanism of artemisinin and its derivatives are not definitely clear. The metabolite M1 was the first active metabolite discovered in red blood cells, where is the actual site the parasites live and the drug works. Therefore, it is significant for developing novel active metabolite agents and helpful for further antimalarial mechanism research. In particular, the microbial transformation provided a green and powerful approach for preparing the active metabolites.

Liquid chromatography mass spectrometry (LC-MS) is a powerful method for the analysis of compounds, and is increasingly used as a sensitive and efficient tool for metabolite profiling in natural plants or bodies. The numerical results of response, closely related to the conversion rate of the transformation products, were meaningful to guide for isolation work. Herein, UPLC-Q-TOF-MS^E^ was employed for predicting and identifying biotransformation products of DHA. Although there were nine compounds discovered and analyzed by MS^E^, only compounds **M1**, **M8,** and **M9**, the top three high response values, were gained by isolation. Therefore, how to increase the conversion rate from substrates to hydroxylation products is one of the significant for further research. Additionally, to satisfy the need of microbial transformation, the purity of DHA was detected, and the HPLC profile of DHA has been shown in Supplementary Figure S2. The two isoforms, *α*-DHA and *β*-DHA, are the two peaks in the graph, respectively.

Artemisinin and its derivatives are excellent antimalarial agents due to the unique peroxide bridge. The mechanism of antimalarial action research indicated that the hallmark of artemisinin activation is the generation of highly reactive carbon-centered radicals via endoperoxide cleavage (O'Neill et al., 2010). And 1-deoxidation products were found to be inactive against *P. falciparum* 3D7 strain due to the cleavage of peroxy linkage (Tu, 2011; Kumari et al., 2018; Yang et al., 2020). Furthermore, the *in vitro* experiments verify the above conclusion.

## Conclusion

Microorganisms can mimic mammal metabolism to synthesize metabolites in an environmentally friendly manner. Herein, microbial transformation by *C. elegans*.40250 was first employed as a biotransformation tool to gain metabolites that were discovered in erythrocyte. Finally, nine products were detected from microbial system by UPLC-ESI-Q-TOF-MS^E^, and three intracellular metabolic products were isolated and identified for the first time. Moreover, compound **M1** was a novel antimalarial component, with an antimalarial activity IC_50_ value of 133 nM. In conclusion, the biotransformation of DHA by *Cunninghamella* proved to be an effective procedure for the investigation of metabolism, especially with the advantage of accumulating metabolites, and one intracellular metabolite of DHA with antimalarial activity was the first time to discover.

## Data Availability

The original contributions presented in the study are included in the article/Supplementary Material, further inquiries can be directed to the corresponding authors.

## References

[B1] Asha S. Vidyavathi M. (2009). Cunninghamella - A Microbial Model for Drug Metabolism Studies - A Review. Biotechnol. Adv. 27, 16–29. 10.1016/j.biotechadv.2008.07.005 18775773

[B2] Bai Y. Zhang D. Sun P. Zhao Y. Chang X. Ma Y. (2019). Evaluation of Microbial Transformation of 10-deoxoartemisinin by UPLC-ESI-Q-TOF-MSE. Molecules 24, 3874. 10.3390/molecules24213874 PMC6864820 31661766

[B3] Han F. Lee I.-S. (2016). Microbial Transformation of the Antimalarial Sesquiterpene Endoperoxide Dihydroartemisinin. Nat. Product. Res. 31, 883–889. 10.1080/14786419.2016.1250092 27788595

[B4] Han F. Lee I. S. (2017). Microbial Transformation of the Antimalarial Sesquiterpene Endoperoxide Dihydroartemisinin. Nat. Prod. Res. 31 (8), 883–889. 10.1080/14786419.2016.1250092 27788595

[B5] Hu Y. Highet R. J. Marion D. Ziffer H. (1991). Microbial Hydroxylation of a Dihydroartemisinin Derivative. J. Chem. Soc. Chem. Commun. 17, 1176–1177. 10.1039/C39910001176

[B6] Kreidenweiss A. Kremsner P. G. Mordmüller B. (2008). Comprehensive Study of Proteasome Inhibitors against Plasmodium Falciparum Laboratory Strains and Field Isolates from Gabon. Malar. J. 7, 187. 10.1186/1475-2875-7-187 18816382 PMC2569964

[B7] Kumari A. Karnatak M. Singh D. Shankar R. Jat J. L. Sharma S. (2018). Current Scenario of Artemisinin and its Analogues for Antimalarial Activity. Eur. J. Med. Chem. 163, 804–829. 10.1016/j.ejmech.2018.12.007 30579122

[B8] Lee I.-S. ElSohly H. N. Croom E. M. Hufford C. D. (1989). Microbial Metabolism Studies of the Antimalarial Sesquiterpene Artemisinin. J. Nat. Prod. 52 (2), 337–341. 10.1021/np50062a020 2746260

[B9] Li Y.-N. Fan M.-L. Liu H.-Q. Ma B. Dai W.-L. Yu B.-Y. (2019). Dihydroartemisinin Derivative DC32 Inhibits Inflammatory Response in Osteoarthritic Synovium through Regulating Nrf2/NF-Κb Pathway. Int. Immunopharmacology 74, 105701. 10.1016/j.intimp.2019.105701 31228817

[B10] Ma Y. Sun P. Zhao Y. Wang K. Chang X. Bai Y. (2019a). A Microbial Transformation Model for Simulating Mammal Metabolism of Artemisinin. Molecules 24, 315. 10.3390/molecules24020315 PMC6358782 30654552

[B11] Ma Y. Zhu Y. Zhang D. Meng Y. Tang T. Wang K. (2019b). Eco-friendly Decarboxylative Cyclization in Water: Practical Access to the Anti-malarial 4-quinolones. Green. Chem. 21, 478–482. 10.1039/C8GC03570A

[B12] O’Neill P. M. Barton V. E. Ward S. A. (2010). The Molecular Mechanism of Action of Artemisinin-The Debate Continues. Molecules 15 (3), 1705–1721. 10.3390/molecules15031705 20336009 PMC6257357

[B13] Parshikov I. A. Muraleedharan K. M. Avery M. A. Williamson J. S. (2004). Transformation of Artemisinin by *Cunninghamella Elegans* . Appl. Microbiol. Biotechnol. 64 (6), 782–786. 10.1007/s00253-003-1524-z 14735322

[B14] Reiter C. Fröhlich T. Gruber L. Hutterer C. Marschall M. Voigtländer C. (2015). Highly Potent Artemisinin-Derived Dimers and Trimers: Synthesis and Evaluation of Their Antimalarial, Antileukemia and Antiviral Activities. Bioorg. Med. Chem. 23 (17), 5452–5458. 10.1016/j.bmc.2015.07.048 26260339

[B15] Tu Y. (2016). Artemisinin-A Gift from Traditional Chinese Medicine to the World (Nobel Lecture). Angew. Chem. Int. Ed. 55, 10210–10226. 10.1002/anie.201601967 27488942

[B16] Tu Y. (2011). The Discovery of Artemisinin (Qinghaosu) and Gifts from Chinese Medicine. Nat. Med. 17, 1217–1220. 10.1038/nm.2471 21989013

[B17] Tu Y. Y. (2009). “Dihydroartemisinin,” in Artemisinin and Artemisinin Drugs (Beijing, China: Beijing Chemical Industry Press), 187–191.

[B18] Wang D. Zhong B. Li Y. Liu X. (2018). Dihydroartemisinin Increases Apoptosis of colon Cancer Cells through Targeting Janus Kinase 2/signal Transducer and Activator of Transcription 3 Signaling. Oncol. Lett. 15, 1949–1954. 10.3892/ol.2017.7502 29434895 PMC5776939

[B19] Wang X. Xu C. C. Liu J. H. (2013). Optimization of the Biotransformation Medium of Dihydroartemisinin. Nat. Prod. Res. Dev. 25, 1690–1695. 10.14233/ajchem.2013.oh20

[B20] World Health Organization (2020). World Malaria Report. Available at: https://apps.who.int/iris/handle/10665/330011 .

[B21] Yang J. He Y. Li Y. Zhang X. Wong Y.-K. Shen S. (2020). Advances in the Research on the Targets of Anti-malaria Actions of Artemisinin. Pharmacol. Ther. 216, 107697. 10.1016/j.pharmthera.2020.107697 33035577 PMC7537645

[B22] Yang L. Zhang D. (2017). Summary of Dihydroartemisinin and its Application for the Treatment of Lupus Erythematosus. Chin. Sci. Bull. 62, 2007–2012. 10.1360/N972017-00172

[B23] Zhan Y. Wu Y. Xu F. Bai Y. Guan Y. Williamson J. S. (2017). A Novel Dihydroxylated Derivative of Artemisinin from Microbial Transformation. Fitoterapia 120, 93–97. 10.1016/j.fitote.2017.05.015 28576722

[B24] Zhang T. Zhang Y. Jiang N. Zhao X. Sang X. Yang N. (2020). Dihydroartemisinin Regulates the Immune System by Promotion of CD8+ T Lymphocytes and Suppression of B Cell Responses. Sci. China Life Sci. 63, 737–749. 10.1007/s11427-019-9550-4 31290095

[B25] Zhao Y. Sun P. Ma Y. Chang X. Chen X. Ji X. (2021). Metabolite Profiling of Dihydroartemisinin in Blood of Plasmodium-Infected and Healthy Mice Using UPLC-Q-TOF-MSE. Front. Pharmacol. 11, 614159. 10.3389/fphar.2020.614159 33536920 PMC7848114

