# Supplementary figures and images for: A Novel Antimalarial Metabolite in Erythrocyte From the Hydroxylation of Dihydroartemisinin by Cunninghamella elegans
Source: Front Chem. 2022 Apr 26;10:850133. doi: 10.3389/fchem.2022.850133 (PMC9086495; doi:10.3389/fchem.2022.850133)

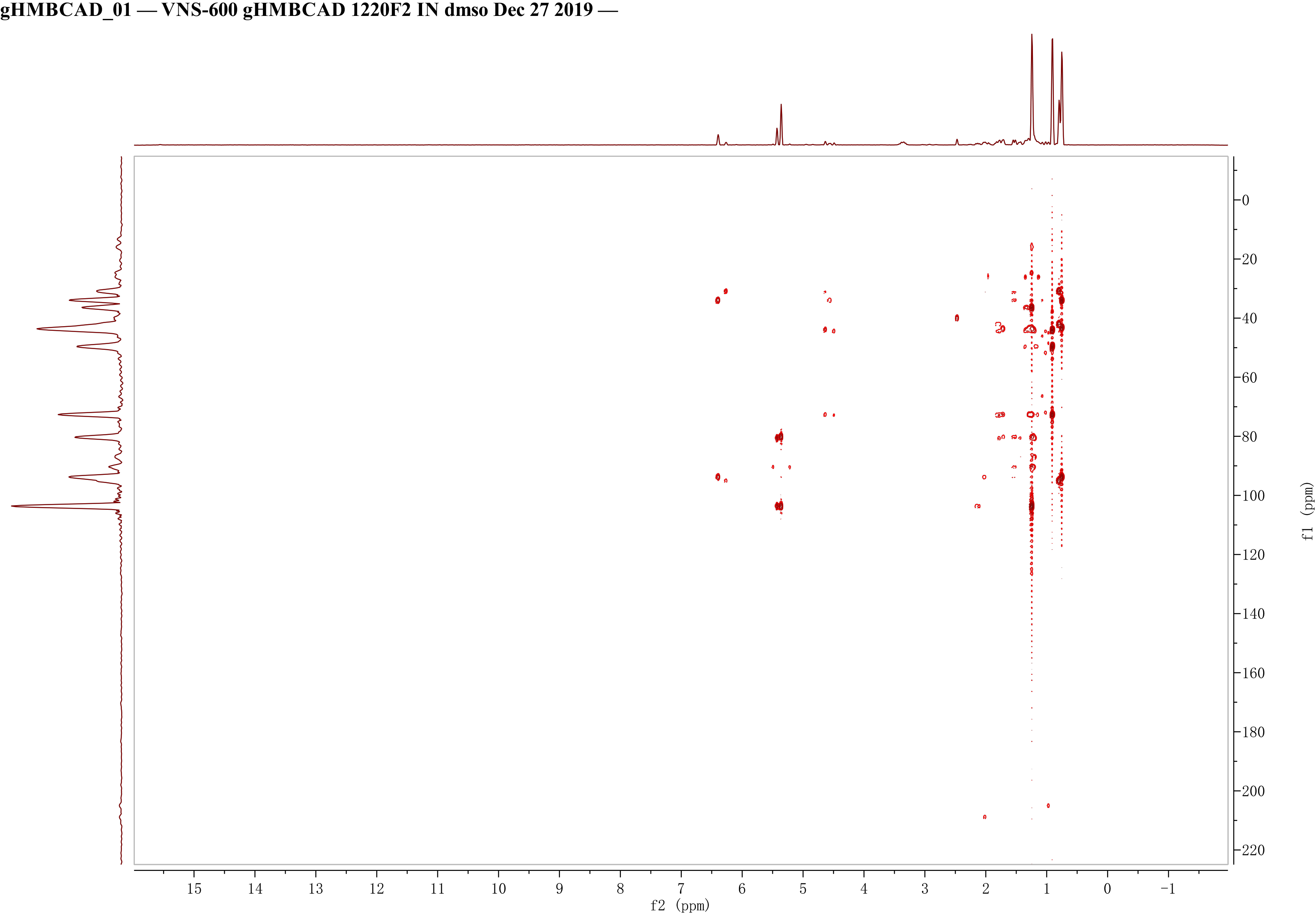

Supplement: Supplementary file 1 [file Image1.tif]
