# Supplementary material for: A Novel Antimalarial Metabolite in Erythrocyte From the Hydroxylation of Dihydroartemisinin by Cunninghamella elegans
Source: Front Chem. 2022 Apr 26;10:850133. doi: 10.3389/fchem.2022.850133 (PMC9086495; doi:10.3389/fchem.2022.850133)
Supplement: Supplementary file 2 [file DataSheet1.PDF]

Supplementary Material

1 Supplementary Figures

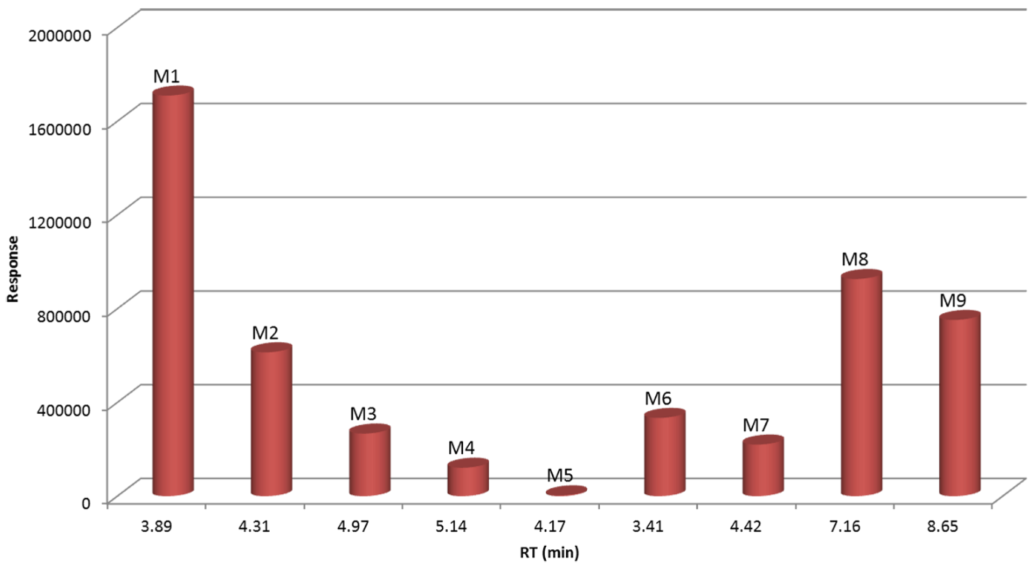

Figure S1. The response strength of dihydroartemisinin products

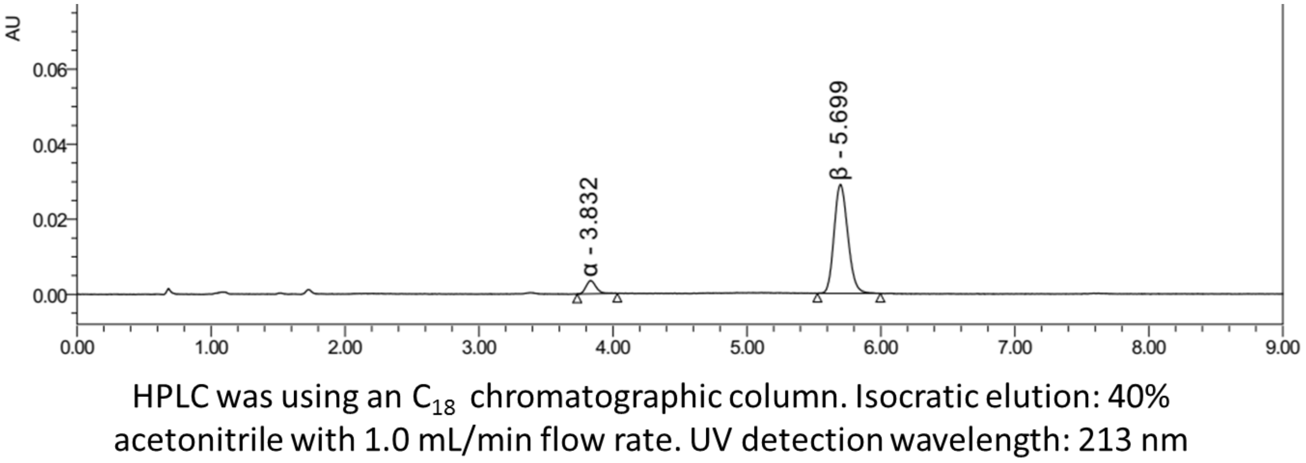

Figure S2. The HPLC profile of Dihydroartemisinin
